# Comparison of Posterior and Anterolateral Surgical Approaches in Treating Adult Humeral Shaft Fractures

**DOI:** 10.7759/cureus.39755

**Published:** 2023-05-30

**Authors:** Mustafa Çukurlu, Ozan Keçeli, İsmail Ağır

**Affiliations:** 1 Department of Orthopaedics and Traumatology, Adıyaman University Training and Research Hospital, Adıyaman, TUR

**Keywords:** radial nerve palsy, postoperative complications, posterior approach, anterolateral approach, humeral shaft fracture

## Abstract

Aim: The purpose of this study is to compare the outcomes and complications of two different surgical approaches, the anterolateral and posterior approaches, for treating humeral shaft fractures.

Materials and methods: Between January 2015 and May 2021, 51 patients with humeral shaft fractures were treated with anterolateral and posterior approaches. Twenty-nine patients were operated with the posterior approach (group 1) and 22 with the anterolateral approach (group 2). Statistical analyses were performed between the two groups regarding age, gender distribution, fractured side, body mass index (BMI), type of trauma, Arbeitsgemeinschaft für Osteosynthesefragen/Orthopaedic Trauma Association (AO/OTA) classification, and follow-up time. Complications such as operative time, amount of bleeding, incision length and implant fracture, radial nerve palsy, wound infection, and nonunion were compared between the two groups. Functional results of the elbow joint were evaluated with the Mayo Elbow Performance Score.

Results: The mean follow-up period was 49.10±21.15 months (12-75 months) in group 1 and 50.00±23.71 months (range: 15-70 months) in group 2. There was no statistical difference between the groups in terms of age, gender distribution, fractured side, BMI, trauma type, AO/OTA classification, and follow-up time (p>0.05). There was no significant difference between the two groups in terms of operation time, intraoperative bleeding, and incision length (p>0.05). The mean Mayo Elbow Performance Score was 77.24±20.03 (range: 70-100 points) in group 1 and 81.36±8.34 (range: 70-100 points) in group 2, and no significant difference was found (p>0.05). When evaluated in terms of complications, there was no significant difference between the groups (p>0.05). While there was no significant difference between the two groups regarding elbow joint range of motion, the limitation was observed in more patients in group 1.

Conclusion: Similar satisfactory treatment results were obtained in patients who underwent anterolateral and posterior approaches in treating humeral shaft fractures. Furthermore, no difference was found between the two approaches regarding complication rates.

## Introduction

The incidence of humeral shaft fractures is 3-5% and shows a bimodal distribution. It usually occurs due to high-energy trauma in young people and low-energy trauma due to osteopenia in older ages [1]. Bergdahl et al. reported that humeral fractures are common, but the relationship between pathoanatomical fracture patterns and patient characteristics has yet to be adequately studied, and epidemiological information is scarce [2]. The injury mechanism of humeral shaft fractures occurs as a result of high-energy traumas such as traffic accidents in young people. In the elderly, it occurs with low energy, such as a simple fall [1]. Humeral fractures are classified based on the Arbeitsgemeinschaft für Osteosynthesefragen (AO) classification, according to the fracture type and location. In the Arbeitsgemeinschaft für Osteosynthesefragen/Orthopaedic Trauma Association (AO/OTA) classification system, fractures are divided into three main types according to their morphology: type A (simple fracture line), type B (separate wedge fragment), and type C (segmental fracture). All main types are divided into groups: type A in spiral, oblique, and transverse fractures; type B in intact and fragmented wedge fractures; and type C in intact and fragmented segmental fractures. Moreover, fractures have qualifications according to the location of the center of the fracture.

Conservative and surgical methods are used to treat humeral shaft fractures [3]. Conservative treatment options are velpau bandage, hanging cast, U splint, functional bracing, and Sarmiento cast. Excellent functional results can be obtained with functional bracing in middle humeral shaft fractures. However, complications such as long-term immobilization, skin problems, fracture malposition, and limitation of joint movement may be seen [4]. Operative therapy can be considered for early active movement and in case of nonunion after conservative treatment. Absolute surgical indications are an open fracture, a vascular injury requiring repair, a brachial plexus injury, an ipsilateral forearm fracture (floating elbow), and periprosthetic shaft fractures at the tip of the stem. Relative indications are pathological fractures, bilateral humerus fractures, burns or soft tissue injury that precludes bracing, multiple trauma, or related lower-extremity fractures.

Open reduction and compression plating remain the treatment for humeral shaft fractures. Plating of humeral shaft fractures has been advocated as the surgical treatment of choice, as it has been shown to cause less reoperation and shoulder impingement compared to intramedullary nailing [5]. Recent reports of minimally invasive plate osteosynthesis for humeral shaft fractures have demonstrated a good range of motion in adjacent joints. The goal should be uneventful fracture healing while preservation of alignment, absence of pain, and return to the full range of motion [5]. During surgery, it is important to be aware of the danger zone for the radial nerve. This area is about three to five-eighths from the tip of the acromion process to the lateral epicondyle when the humeral length is divided into eight parts [6].

Although the anterolateral and posterior approaches vary according to the surgeon's preference and experience, they are the most commonly used approaches in treating humeral shaft fractures [7].

This study compares treatment results and complication rates between anterolateral and posterior surgical humeral approaches in managing humeral shaft fractures.

## Materials and methods

Study design

This retrospective study was performed in the Department of Orthopaedics and Traumatology, Adiyaman University Training and Research Hospital. The study followed the principles outlined in the Declaration of Helsinki and was approved by the Institutional Review Board (approval number: 2022/7-51). Patients operated on between January 2015 and May 2021 for a humerus shaft fracture with a fracture line between the surgical neck distal and proximal to the supracondylar region were retrospectively reviewed. Inclusion criteria included closed humeral shaft fractures over 18 years of age with postoperative 1-day, 1-month, 6-month, and 12-month radiographs. Patients with pathological and open fractures, fractures involving the elbow or shoulder joint with the humerus shaft, patients with a previous surgical history, patients who underwent intramedullary nailing or minimally invasive percutaneous plate osteosynthesis (MIPPO), patients with no regular follow-up or less than one-year follow-up, and additional injuries were excluded from the study.

Radiographs were taken on the first day after surgery. The patient began doing elbow and shoulder movements, both active and passive, on the second day following the surgery. Patients were scheduled for check-ups every four weeks during the first three months and then once a month afterward. In these controls, the patients were requested to have X-rays. Union was mentioned when cal tissue was seen in all three cortices in the control radiographs [8]. Patients who experienced a limited range of motion in their shoulder or elbow joints were referred to the physical therapy and rehabilitation center after one month. The patients' Mayo elbow performance score was used, consisting of four components: Pain, Movement, Stability, and Function; the maximum possible score is 100. The Mayo elbow performance score is a reliable and valid tool for assessing elbow function [9].

Fifty-one patients who were operated with anterolateral and posterior incisions were included in the study. The surgeries were performed in a single center and by multiple experienced surgeons. Among the patients examined, 29 (Group 1) were operated with the posterior incision and 22 (group 2) with the anterolateral incision. Data such as gender, body mass index, type of trauma, affected side, gender, AO/OTA classification, duration of follow-up, amount of blood loss, mean duration of surgery, length of incision, and Mayo score were collected.

**Figure 1:**
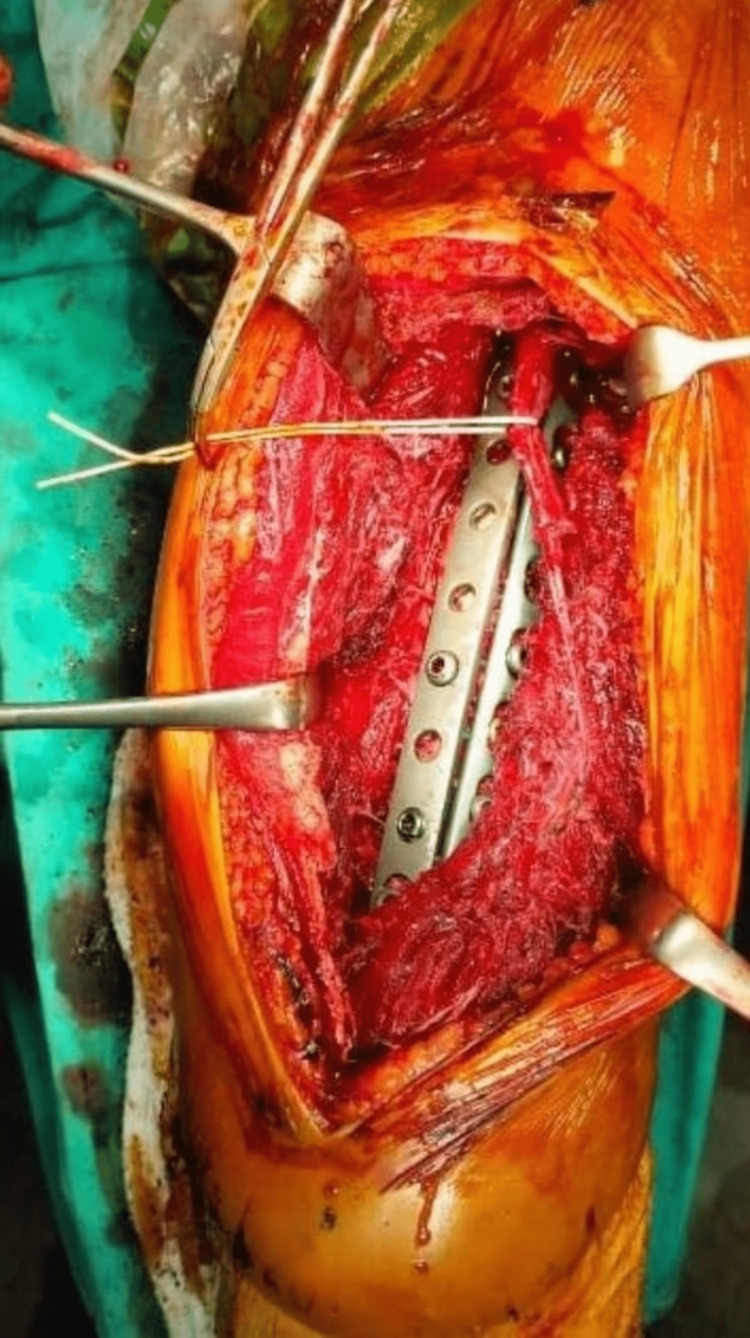
Surgical view of the posterior approach

Group 1 patients operated with a posterior incision were placed in the lateral decubitus position with the fractured side up. The arm was completely free and placed on the arm holder, and it was prepared sterile without applying a tourniquet. The incision was made by centering the fracture line by taking the posterolateral corner of the acromion in the proximal and the olecranon in the distal. After superficial exposure, the triceps was divided into two by sharp dissection (Figure 1). The radial nerve and the brachial profunda artery were dissected and preserved [10].

**Figure 2:**
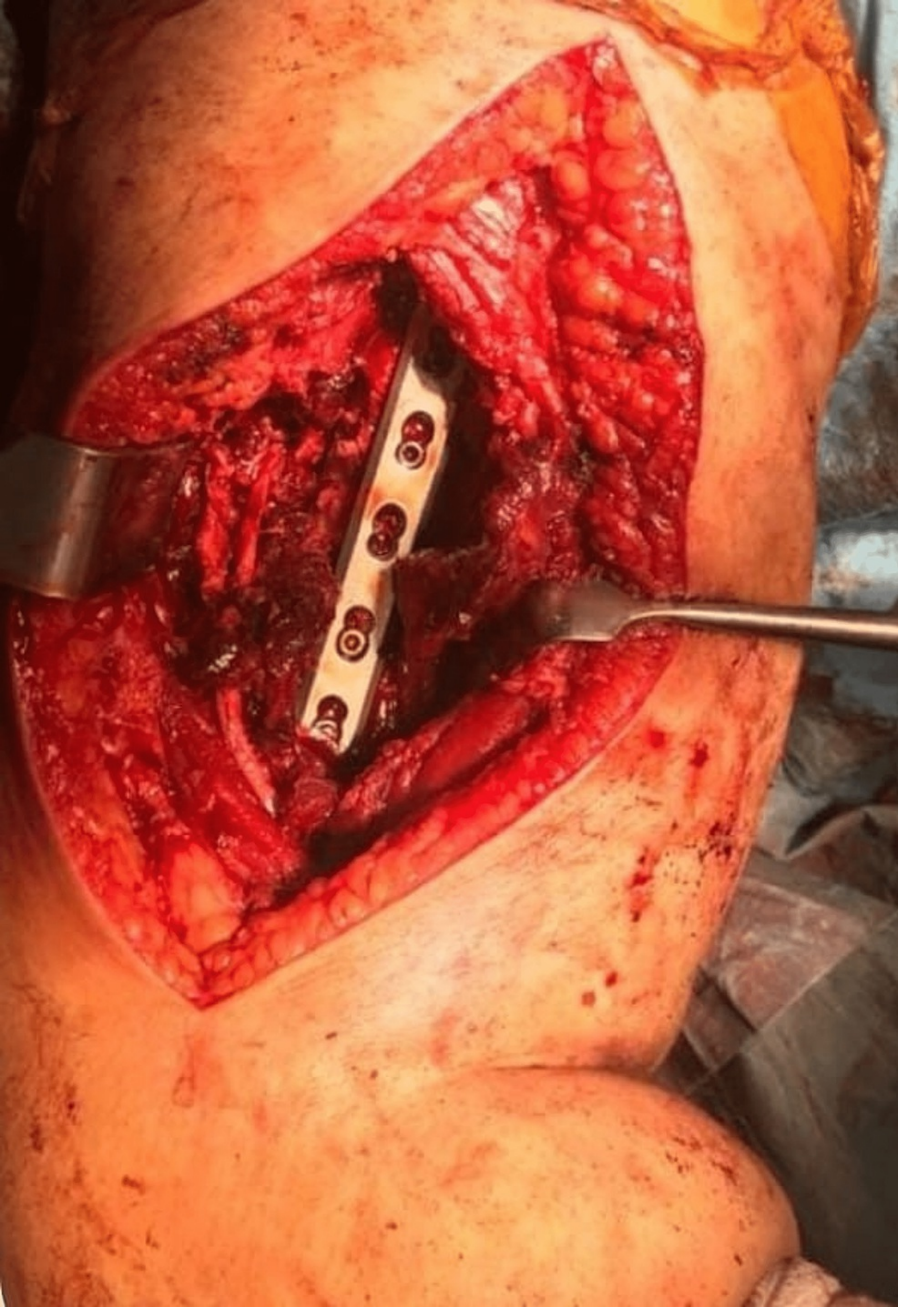
Surgical view of the anterolateral approach

Group 2 patients with an anterolateral incision were placed in a semi-sitting (chaise lounge) position. Similarly, it was prepared sterile without applying a tourniquet. The coracoid process proximally and the lateral supracondylar region distally were guided so that the incision fracture line was centered. The lateral border of the biceps muscle was palpated, the incision was started, and a superficial dissection was performed. Dissection was continued between the biceps and triceps muscles, and the brachialis muscle was dissected longitudinally to reach the bone and fracture line (Figure 2). Dissection proceeds distally anterior to the lateral supracondylar region, and medially the brachialis is located laterally. In this region, the radial nerve enters the surgical area by circulating lateral to the distal humerus, and it was explored and preserved proximally in this area [11].

Choosing a dynamic compression plate or a locked angle plate and placing these plates on the anterolateral or anteromedial surface of the humerus was at the surgeon's discretion. Lag screws were used to achieve an anatomical reduction in oblique fractures or butterfly fragments. Fixation was performed with three screws to the distal and proximal of the fracture.

Statistical analysis

We used IBM SPSS 23.0 software from IBM Corp. in Armonk, NY, USA, to analyze the data. Descriptive statistical methods were applied to summarize the data. The Shapiro-Wilk test was used to evaluate the normality of continuous variables, and Pearson chi-square independence tests were used to assess independence between two categorical variables. We used Mann-Whitney U-test and Kruskal-Wallis test to compare data that did not show normal distribution. Chi-square tests of independence were employed to investigate relationships between classified variables in 2x2 crosstabs. A p-value of less than 0.05 was considered statistically significant.

## Results

Patient demographics are shown in Table 1. There was no difference between the groups regarding age, gender, fracture site, body mass index, mechanism of injury, and AO/OTA classification.

**Table 1:** Demographic data of patients AO/OTA: Arbeitsgemeinschaft für Osteosynthesefragen/Orthopaedic Trauma Association

Demographic data		Type of incision		p-Value
		Posterior approach (n=29)	Anterolateral approach (n=22)	
Age		45.48±20.38	46.14±16.25	0.718
Gender	Female	11	11	0.410
	Male	18	11	
Fracture side	Right	12	10	0.784
	Left	17	12	
Body mass index		26.05±3.53	27.81±6.10	0.234
Trauma	Fall	20	15	0.925
	Traffic accident	6	4	
	Sports injury	3	3	
AO/OTA classification	A	17	8	0.122
	B	11	10	
	C	1	4	
Follow-up time (month)		49.10±21.15	50.00±23.71	0.895

There was no statistically significant difference between the groups in the amount of blood loss during surgery, incision length, and Mayo scores (Table 2).

**Table 2:** Relationship between incision types and amount of blood loss, surgery time, length of incision, Mayo score

Parameters	Type of incision		p-Value
	Posterior (n=29)	Anterolateral (n=22)	
Amount of blood loss (mL)	197.41±31.21	192.73±30.03	0.675
Operation time	86.00±6.81	85.32±7.96	0.497
Incision length (cm)	18.69±2.95	20.05±3.06	0.107
Mayo score	77.24±20.03	81.36±8.34	0.984

Postoperative complications are shown in Table 3. There was no significant difference between the groups in terms of complications. Radial nerve lesions resolved spontaneously in four patients with wrist extension loss in the first five months of postoperative follow-up and in one patient with mild sensory loss in the hand's radial nerve territory at seven months. Patients with implant failure and nonunion were revised, and the complete union was achieved. Superficial or deep infection was not seen in any patient. Patients with limited elbow range of motion (ROM) were included in the physical therapy program. Nearly complete ROM was obtained in three of the six patients operated with posterior incisions, and one operated with the lateral incision.

**Table 3:** Relationship between incision types and postoperative complications ROM: range of motion

Postoperative complications	Type of incision		p-Value
	Posterior (n=29)	Anterolateral (n=22)	
Implant failure	4	1	0.375
Nonunion	5	1	0.218
Radial nerve palsy	3	1	0.625
Wound infection	0	0	-
Elbow ROM restriction	6	1	0.124

## Discussion

Groups 1 and 2 showed no significant differences in terms of gender, age, body mass index, mechanism of injury, affected side of the fracture, fracture type, follow-up time, amount of blood loss, affected side of the fracture, operation time, incision lengthening, and Mayo score. Although complications such as implant failure, nonunion, radial nerve palsy, and elbow ROM restriction were more common in Group 1 patients, they were not statistically significant.

Humeral shaft fractures are commonly treated with open reduction and internal fixation (ORIF) using plates and screws [12]. The anterolateral approach is widely used by reconstructive surgeons [13]. However, the outcomes of humeral shaft fracture fixation using a medial approach are rarely reported [13]. A retrospective study analyzing surgical techniques for mid-shaft fractures of the humerus found that the anterolateral approach resulted in fewer complications and better functional outcomes than the posterior approach [14].

The use of functional braces in the conservative treatment of humeral mid-shaft fractures can provide excellent clinical outcomes; however, this treatment is associated with various complications, including a high rate of nonunion, which can occur in up to 15% of cases [15]. Surgeons often prefer surgery as a treatment for mid-shaft fractures due to the possibility of a faster recovery of function [16]. Compared to ORIF, the intramedullary nailing technique involves more significant risks of limited shoulder movement and fixation failure. A study examined patients who suffered from diaphyseal fractures of the humerus and received either ORIF or MIPPO treatment. The study found no notable differences in functional outcomes or complications between the two treatment options [17]. According to reports, patients undergoing MIPPO treatment may experience higher rates of mal-rotation and mal-union, leading to shoulder joint degeneration over time. There is some debate about which method is better, but ORIF is generally considered a more dependable osteosynthesis technique [18].

In our study, although we found more complications in patients treated with the posterior approach, no statistically significant difference was found. A posterior approach (triceps separation or triceps reflecting) is recommended for fractures of the middle one-third of the humerus [19]. Many retrospective studies have found 92% to 100% union rates with the posterior approach [20,21]. The recent advantages of the posterior approach are the direct visualization of the radial nerve and its appropriateness for plate fixation of the posterior humerus [20]. The lateral or prone position used in the posterior approach may pose a potential danger in anesthesia patients with multi-trauma. The anterolateral approach can be used as an alternative to humeral midshaft fractures. The advantages of the anterolateral approach include the supine position and excellent imaging when the incision is extended proximally and distally [22].

Radial nerve injury is the most common peripheral nerve injury associated with long bone fractures [23]. The incidence of radial nerve palsy in surgery for humeral shaft fractures is between 7% and 17%. In another multicenter retrospective study in which 325 patients were examined, they found an average of 11% incidence of radial nerve injury after humeral shaft fractures [23]. In a study by Prasarn et al., they stated that the posterior approach carries a risk of nerve damage because the plate must be placed under the radial nerve [24]. In many studies, radial nerve palsy was found to be more common in patients who underwent the posterior approach than in the patients who underwent the anterolateral approach [9,25]. In our study, although the rate of radial nerve palsy was higher in patients operated on with the posterior approach than in the patients who underwent the anterolateral approach, it was not significant. The rate of radial nerve palsy in all patients was consistent with what was reported in the literature.

The treatment of iatrogenic radial nerve palsy is controversial [26]. While most researchers recommend 4-6 months of observation, some recommend early exploration [27]. In one study, neuropraxia was defined as muscle dysfunction without atrophy, complete loss of sensation in the area innervated by the radial nerve, and absence of sweating loss [28]. Patients with radial nerve palsy mainly developed neuropraxia, which improved during follow-up. However, when other studies are reviewed, immediate surgical intervention is recommended in patients with a strong Tinel sign with complete sensory and motor deficits [29]. In our study, sweating and complete sensory loss were not detected in patients with radial nerve palsy. Three patients with radial nerve palsy recovered entirely in the first five months after surgery, and one patient in the seventh month.

Studies have shown no significant difference in union rates between patients who underwent posterior and anterolateral approaches in the surgical treatment of humeral shaft fractures [15]. However, the optimal plating approach remains an open question that needs to be validated by biomechanical studies [14]. Although the nonunion rate was higher in patients who underwent a posterior approach in our study, it was not statistically significant. There is no specific study comparing wound infection in patients who underwent posterior and anterolateral approaches in the surgical treatment of humeral shaft fractures. No meaningful information could be obtained in our patient groups.

The Mayo elbow performance score is a widely used scoring system to evaluate elbow function after surgical treatment of humeral shaft fractures [22]. According to a study by Li et al., they did not find a significant difference between patients who underwent posterior and anterolateral approaches [16]. In our study, elbow ROM limitation was higher in patients who underwent a posterior approach, and the Mayo score was lower, but it was not significant.

The limitations of our study are that it is retrospective, heterogeneous, and has relatively small patient groups. While there was a variation in radial nerve palsy and nonunion occurrences between the groups, the difference was not considered statistically significant due to the small sample size. A multicenter prospective randomized controlled trial should be conducted to confirm our findings.

## Conclusions

Various surgical approaches are recommended for humeral shaft fractures. The literature reports no significant difference between posterior and anterolateral approaches regarding clinical, radiographic, and radial nerve palsy.

In the surgical treatment of humeral shaft fractures, satisfactory results were obtained in both anterolateral and posterior surgical approaches compared to our study. We have found that both approaches are safe. The surgeon's approach to use depends on his knowledge and experience.
